# Apigenin activates AKT1 to coordinate MTOR-mediated anti-apoptosis and FOXO3-driven antioxidant defense in calcium oxalate nephropathy

**DOI:** 10.1097/JS9.0000000000005023

**Published:** 2026-03-12

**Authors:** Lei Li, Xiong Xu, Linfa Guo, Tongzu Liu

**Affiliations:** a Department of Urology, Zhongnan Hospital of Wuhan University, Wuhan, China; b Hubei Key Laboratory of Urological Diseases, Wuhan, China; c Hubei Clinical Research Center for Laparoscopic/Endoscopic Urologic Surgery, Wuhan, China; d Institute of Urology, Wuhan University, Wuhan, China; e Hubei Medical Quality Control Center for Laparoscopic/Endoscopic Urologic Surgery, Wuhan, China; f Wuhan Clinical Research Center for Urogenital Tumors, Wuhan, China

**Keywords:** anti-apoptosis, anti-oxidative stress, apigenin, CaOx crystal-induced kidney injury, signaling pathway

## Abstract

**Background:**

Kidney stone disease, primarily composed of calcium oxalate (CaOx) crystals, represents a significant global health burden with high recurrence rates. Current therapeutic strategies fail to adequately address the oxidative stress and apoptosis triggered by CaOx crystals in renal tubular cells. While the natural flavonoid apigenin (API) shows promise, its precise mechanism in CaOx nephropathy remains unclear.

**Methods:**

We employed an integrated strategy combining network pharmacology and machine learning to identify potential therapeutic targets of apigenin in kidney stone disease. Experimental validation was conducted using both *in vivo* and *in vitro* models: a mouse model of CaOx nephropathy induced by glyoxylate and HK-2 cells exposed to calcium oxalate monohydrate crystals. The interaction between apigenin and AKT1 was investigated through surface plasmon resonance, molecular docking, cellular thermal shift assays, and drug affinity responsive target stability assays. Downstream signaling effects were analyzed using quantitative PCR, Western blotting, reactive oxygen species measurement, and apoptosis assessment. The functional role of AKT1 was further examined using the specific inhibitor MK-2206, siRNA-mediated knockdown, and FOXO3 overexpression experiments.

**Results:**

Apigenin treatment significantly reduced CaOx crystal deposition, improved renal function markers, and attenuated tubular damage in mice in a dose-dependent manner. Bioinformatic analysis identified AKT1 as the core target, and experimental validation confirmed that apigenin directly binds to AKT1 with high affinity, leading to enhanced phosphorylation. This activation modulated two critical downstream pathways. The mTOR pathway suppressed apoptosis through downregulation of BAX and upregulation of BCL-2; Phosphorylation of FOXO3 decreased its transcriptional activity on Keap1, resulting in reduced Keap1 expression, subsequent Nrf2 stabilization, nuclear translocation, and upregulation of the antioxidant enzyme HO-1. The essential role of AKT1 was further supported by the finding that MK-2206 and si-AKT1 treatment abolished apigenin’s protective effects. Additionally, FOXO3 overexpression reversed apigenin-induced Nrf2 activation, confirming the involvement of the AKT1-FOXO3-Keap1-Nrf2 axis.

**Conclusion:**

Apigenin alleviates CaOx-induced renal injury through direct activation of AKT1, which coordinates dual protective mechanisms. Anti-apoptosis via the mTOR pathway and antioxidant defense through the FOXO3-Keap1-Nrf2 axis. This study provides comprehensive evidence of apigenin’s AKT1-centered mechanism of action in CaOx nephropathy, supporting its potential as a novel therapeutic agent for kidney stone disease.

## Introduction

Kidney stones disease (KSD), or nephrolithiasis represents a prevalent disorder affecting the urinary tract worldwide, with a continuously increasing incidence rate of 14.8% per year^[^1^]^. Epidemiological surveys show that the prevalence of this disease is 1–5% in Asia, 5–9% in Europe, and as high as 7–13% in North America^[^2^]^. From component analysis, about 80% of kidney stones are composed of calcium oxalate (CaOx), mainly in the form of calcium oxalate monohydrate (COM) and calcium oxalate dihydrate (COD)^[^3^]^. Although modern urological techniques such as extracorporeal shock wave lithotripsy (ESWL) and ureteroscopy have significantly improved the treatment of stones^[^4^]^, the disease still faces two major clinical challenges: first, a recurrence rate of up to 50% within 5–10 years after surgery; Secondly, it may lead to serious complications such as chronic kidney disease (CKD)^[^5^]^. The existence of these issues highlights the necessity of in-depth research on the pathogenesis of kidney stones.

In recent years, the research focus has gradually shifted towards the molecular mechanism of kidney stone formation. Numerous pieces of evidence indicate that the imbalance of oxalic acid steady state can trigger hyperoxaluria or CaOx crystals can lead to damage and apoptosis of human renal proximal tubular epithelial (HK-2) cells, thereby disrupting the reabsorption and secretion functions of renal tubules and promoting supersaturation of stone forming substances in urine^[^6,7^]^. In addition, the damaged HK-2 cell surface provides favorable conditions for the attachment and growth of CaOx crystals^[^8,9^]^. These findings provide a new perspective for a deeper understanding of the pathogenesis of kidney stones, and also point the way for the development of new prevention and therapeutic modalities. Therefore, the systematic study of the molecular mechanism of kidney damage not only has important theoretical value, but also has the potential to provide new intervention targets for clinical prevention and treatment. Current strategies for preventing kidney stone recurrence primarily involve dietary modifications (such as increasing fluid intake and limiting oxalate consumption), thiazide diuretics, and alkali citrate therapy^[^10^]^. However, these approaches often have limited efficacy, poor long-term adherence, and inadequate targeting of the underlying cellular damage, particularly the oxidative stress and apoptosis triggered by CaOx crystals in renal tubular cells. The need for agents that can directly mitigate these pathological processes while offering favorable safety and bioavailability remains unmet. Natural flavonoids have emerged as promising candidates due to their multi-target potential and low toxicity profile^[^11^]^.

Apigenin (4′,5,7-trihydroxyflavone, API) is a naturally occurring flavonoid compound that is abundant in various fruits (such as citrus), vegetables (such as celery, onions), and herbs, with low toxicity and good bioavailability^[^12–14^]^. More and more evidence suggests that API have multiple pharmacological properties, particularly significant antioxidant, anti-inflammatory, and anti-apoptotic activities, indicating their therapeutic potential for various pathological conditions^[^15,16^]^. It is worth noting that API exerts strong antioxidant effects by clearing free radicals and inhibiting the progressive reactive oxygen species (ROS) deposition, effectively reducing oxidative stress-induced damage. For example, in an animal model of Alzheimer’s disease induced by aluminum chloride induced neurotoxicity, API has been shown to alleviate oxidative stress, neuroinflammation, and cognitive impairment^[^17^]^. In addition, API can also affect the process of cell apoptosis by regulating multiple key signaling pathways, and API exhibit context dependent regulation of apoptosis pathways across different tissues. In the Parkinson’s disease model, API exhibits neuroprotective effects by inhibiting cell apoptosis induced by alpha synuclein^[^18^]^, while in turn promoting apoptosis of pancreatic stellate cells^[^19^]^. These findings highlight the ability of API to regulate oxidative stress and apoptosis pathways, emphasizing their potential therapeutic value in organ protection, tumor intervention, and metabolic disorders. While previous studies have reported the antioxidant and anti-apoptotic properties of apigenin in various disease models, including urolithiasis, its precise molecular targets and the signaling network through which it exerts its renoprotective effects in CaOx nephropathy remain poorly defined^[^20^]^. In particular, whether apigenin coordinately regulates anti-apoptotic and antioxidant pathways through a central signaling hub remains unexplored. Employing an integrated approach combining network pharmacology, machine learning, and experimental validation, we identified AKT1 as the core target of apigenin in kidney stone disease. Diverging from prior studies focusing on isolated pathways, our work demonstrates that apigenin directly binds and activates AKT1, thereby concurrently modulating the mTOR-mediated anti-apoptotic pathway and the FOXO3/Nrf2-driven antioxidant axis. This dual-pathway coordination establishes a novel mechanistic framework for the renoprotective effects of apigenin and underscores AKT1 as a pivotal therapeutic target in CaOx nephropathy, offering a more comprehensive strategy compared to single-pathway interventions. The aim of this study is to elucidate the protective mechanism of API against calcium oxalate crystal induced renal injury and provide scientific evidence for its potential clinical applications. This work has been reported according to TITAN standards and cited in the references^[^21^]^.

HIGHLIGHTS

- For the first time, apigenin was found to regulate the mTOR/FOXO3 dual pathway by targeting AKT1, while inhibiting cell apoptosis and oxidative stress.
- Integrating network pharmacology and machine learning algorithms to establish a target prediction system with AKT1 as the core, providing a new paradigm for natural medicine research.
- It has been confirmed that apigenin can significantly reduce the deposition of calcium oxalate crystals and has good safety, providing a potential clinical solution for the prevention and treatment of kidney stones.

## Material and methods

### Construction and administration method of calcium oxalate crystal kidney mouse model

A total of 35 eight-week-old male C57BL/6J mice, purchased from Shulaibao Biotechnology Co., Ltd. (Wuhan, China), were housed under standard conditions. After acclimatization, they were randomly allocated into seven experimental groups (*n* = 5 per group) using a random number table: (1) blank control group; (2) vehicle group; (3) glyoxylate (Gly) group; (4) Gly + N-acetylcysteine (NAC); (5) Gly + apigenin (10 mg/kg/d, L-API); (6) Gly + apigenin (25 mg/kg/d, M-API); and (7) Gly + apigenin (50 mg/kg/d, H-API). In the subsequent mechanistic validation phase, 20 eight-week-old male C57BL/6J mice were similarly randomized into four experimental groups (*n* = 5 per group): (1) Control; (2) Gly; (3) Gly + API (50 mg/kg/d); and (4) Gly + API + MK-2206. Apigenin was suspended in 0.5% sodium carboxymethyl cellulose (CMC-Na) and administered orally once daily for 10 consecutive days, with mice fasted for 4 hours prior to each administration^[^22^]^. The vehicle group received 0.5% CMC-Na. To validate the AKT1-dependent mechanism, the AKT1 inhibitor MK-2206 (MedChemExpress, USA) was dissolved in phosphate-buffered saline (PBS) and administered intraperitoneally at a dose of 40 mg/kg/d, 1 hour prior to apigenin treatment in the relevant groups^[^23^]^. From day 4 to day 10, all treatment groups received intraperitoneal injections of glyoxylic acid (100 mg/kg/d in PBS) to establish the glyoxylate-induced injury model, while the control group received PBS. In order to standardize absorption and minimize potential interference from food, mice were fasted for 4 hours before each dose of apigenin. On the day following the final glyoxylate injection, orbital blood was collected from all mice under anesthesia for serum biochemical analysis. Subsequently, the mice were euthanized and kidney specimens were harvested for histological examinations. All procedures complied with the ARRIVE guidelines and were approved by the university’s Institutional Animal Care and Use Committee^[^24^]^. And, apigenin (MCE, USA, chemical structure) shown in the Supplemental Digital Content Figure S1, available at: http://links.lww.com/JS9/H36. The process of animal modeling is described in the Supplemental Digital Content Figure S2, available at: http://links.lww.com/JS9/H37.

### Preparation of Calcium Oxalate Monohydrate (COM) crystals

Mix 10 mmol/l calcium chloride and 1.0 mmol/l sodium oxalate in 10 mmol/l Tris HCL buffer containing 90 mmol/l sodium chloride (pH = 7.4), so that the final concentrations of COM components are 5 mmol/l calcium chloride and 0.5 mmol/l sodium oxalate, respectively, and then incubate overnight at room temperature. The prepared COM was collected by filtration and sterilized under high pressure for subsequent experiments. The final COM crystals were mixed in DMEM/F12 medium to achieve a COM concentration of 10 mg/ml.

### Cell culture and treatment

HK-2 cells were purchased from the China Type Culture Collection Center (Wuhan), and the cell lines were cultured in DMEM/F12 medium (Gibco, USA) containing 10% FBS at 37 °C and 5% CO_2_ in a suitable humidity (Thermo Fisher Scientific, USA) incubator. The cell line has been tested for Mycoplasma and the morphological examination meets the standards of ATCC. When HK-2 cells reached approximately 80% confluence, they were allocated into the following groups: (1) blank control group; (2) vehicle group; (3) COM crystal-treated group; (4) NAC pretreatment + COM group; (5) 25 μM apigenin + COM group; (6) 50 μM apigenin + COM group; and (7) 100 μM apigenin + COM group. In the subsequent mechanistic validation phase, cells were divided into four experimental groups: (1) Control; (2) COM; (3) COM + API; (4) COM + API + MK-2206. MK-2206 was purchased from MCE (HY-108232) and N-acetylcysteine (NAC, HY-B0215) was also sourced from MCE. Specifically, selected groups were pretreated with 100 μM apigenin or 4 mM NAC for 2 hours prior to exposure to COM crystals (2.0 nM), DMSO as solvent control (concentration < 0.1%)^[^22,23^]^. Another group was intervened with 10 μM MK-2206 for 2 hours before apigenin pretreatment^[^25^]^. All cells were co cultured for 24 hours for subsequent analysis.

### Cell transfection

Small interfering RNA targeting human AKT1 (si-AKT1) and a negative control siRNA (si-NC) were designed and synthesized by Tsingke Biotechnology Co., Ltd. The sequence of the AKT1-targeting siRNA was 5′-CUUGGAUUUGUACCAUUCUUC-3′. Cell transfection was performed using Lipofectamine 3000 transfection reagent (Invitrogen, USA). Briefly, HK-2 cells were seeded at an appropriate density in 6-well plates. When cells reached approximately 50% confluence, they were transfected with three different AKT1-specific siRNA sequences (designated #1, #2, and #3) or the negative control siRNA (si-NC) following the manufacturer’s instructions. Based on the transfection treatment, cells were divided into the following groups: Control group, si-NC group, si-AKT1#1 group, si-AKT1#2 group, and si-AKT1#3 group. Cells were harvested 24 hours post-transfection for RNA extraction and 48 hours post-transfection for total protein extraction. Subsequently, Quantitative real-time PCR (qRT-PCR) was performed to detect AKT1 mRNA expression to evaluate the gene knockdown efficiency, and Western blotting was conducted to assess protein-level knockdown efficiency. Furthermore, to establish the FOXO3 overexpression model, the FOXO3 overexpression plasmid was transfected into cells at approximately 50% confluence using Lipofectamine 3000 reagent. After stable transfection, total RNA and protein samples were collected separately, and FOXO3 expression at both mRNA and protein levels was analyzed by qRT-PCR and Western blotting to verify the overexpression efficiency.

### Determination of apigenin concentration in plasma and kidney tissue

Quantitative analysis of apigenin concentration in randomly selected mouse plasma and kidney tissue using a validated high-performance liquid chromatography-tandem mass spectrometry (HPLC-MS/MS) method conducted by Servicebio Biotechnology Co., Ltd. (Wuhan, China). Plasma and kidney homogenates were prepared from samples collected at 0, 1-, 2-, 4-, and 8-hours post-administration. Chromatographic separation was performed on an UltiMate 3000 RS system (Thermo Fisher Scientific) using an Agela Venusil C18 Plus column (50 × 2.1 mm, 5 μm) with a gradient mobile phase of 0.1% formic acid in water and acetonitrile. Chromatographic separation was performed on an UltiMate 3000 RS HPLC system (Thermo Fisher Scientific, USA) equipped with an Agela Venusil C18 Plus column (50 × 2.1 mm, 5 μm). The mobile phase consisted of 0.1% formic acid in water (solvent A) and 0.1% formic acid in acetonitrile (solvent B). A gradient elution program was applied as follows: 0–2.0 minutes, 10% B to 95% B; 2.0–3.8 minutes, 95% B; 3.8–4.0 minutes, 95% B to 10% B; and 4.0–5.0 minutes, 10% B. The flow rate was 0.4 ml/min, and the column temperature was maintained at 35 °C. The injection volume was 2.0 μl. Detection was carried out on a Q Exactive high-resolution mass spectrometer (Thermo Fisher Scientific, USA) with electrospray ionization (ESI) in negative ion mode. The selected reaction monitoring (SRM) transition for apigenin was *m/z* 269.05 → 117.03. The spray voltage was set at 4000 V, capillary temperature at 350 °C, sheath gas pressure at 40 Arb, and auxiliary gas pressure at 10 Arb. A standard curve (0.5–5000 ng/ml) was established using weighted ( $1/X^{2}$) linear regression, exhibiting excellent linearity ( $r^{2}>0.99$).

### Pathological and biochemical testing

The levels of serum creatinine (Cr) and blood urea nitrogen (BUN) in mice are used to reflect renal function. Mouse orbital blood collection was performed, and serum was collected by centrifugation at 3000 r/min for 10 minutes. The operation was carried out according to the instructions of the serum Cr and BUN determination kit. Finally, the absorbance value at a wavelength of 510 nm was measured using an enzyme-linked immunosorbent assay (ELISA) reader. Cr or BUN = sample tube absorbance/standard tube absorbance x standard tube concentration.

Mouse renal tissues were fixed in 4% paraformaldehyde (24 hours) and paraffin-embedded. From these blocks, 4-μm sections were prepared for histological examinations, including periodic acid-Schiff (PAS) and von Kossa staining. Microscopic evaluation was conducted using a Zeiss imaging system. For quantitative assessment, ImageJ software analyzed both calcium oxalate monohydrate (COM) crystal deposition in von Kossa-stained sections and tubular damage percentages in PAS-stained preparations. Apoptosis was evaluated through TUNEL assays following standardized protocols: after dewaxing and antigen retrieval, tissue sections underwent Triton X-100 (0.5%) permeabilization, while cultured cells in 24-well plates were processed similarly before incubation with TdT enzyme and labeling reagents (1 hour, dark conditions). DAPI is used to stain the cell nucleus. Observe the sliced and plated cells using an inverted fluorescence microscope. Apoptosis rate = total cell count (blue)/TUNEL positive cell count (red).

### Cell viability assay

Inoculate HK-2 cells into a 96 well plate, treat with different gradients of apigenin, replace the culture medium with 10% CCK8 reagent (MCE, USA), and incubate for 1 hour. Measure the absorbance at 450 nm using an ELISA reader (Thermo Fisher Scientific, USA). And the changes in cell morphology were measured using an inverted microscope (50×).

### Annexin V-FITC/PI apoptosis assay

According to the manufacturer’s instructions, use the APC Annexin V apoptosis detection kit. Cells from different groups were digested with EDTA free trypsin and washed twice with pre cooled PBS. Then, resuspend the cells with 1× Binding Buffer to a cell concentration of 5 × 10^5^ cells/ml. Stain the cells with 5 μl Annexin V-FITC and 5 μl propidium iodide (PI) in the dark at room temperature for 15 minutes. Add 400 μl of 1× Binding Buffer into each reaction tube to terminate the reaction between reagents. Finally, the apoptosis rate of cells was measured by flow cytometry using Beckmen LX instrument.

### Cell cycle analysis by flow cytometry

To assess the impact of apigenin on cell cycle progression, DNA content was quantified using propidium iodide (PI) staining. Following treatment with various concentrations of apigenin (0, 50, 100, 150, and 200 µM) for 24 hours, HK-2 cells were harvested by trypsinization, washed with cold PBS, and fixed in 70% ice-cold ethanol at 4 °C overnight. After fixation, cells were centrifuged to remove ethanol, washed with PBS, and then incubated in the dark at 37 °C for 30 minutes with 500 µl of staining solution containing PI (50 µg/ml, Sigma-Aldrich) and RNase A (100 µg/ml, Thermo Fisher Scientific) to stain DNA and degrade RNA, respectively. The stained cells were analyzed using Beckman Coulter CytoFLEX LX flow cytometer. For each sample, at least 10 000 single-cell events were collected. The cell cycle distribution was determined using FlowJo v10.0.

### Intracellular reactive oxygen species (ROS) detection

Detection of Mitochondrial ROS in HK-2 Cells Using a ROS Assay Kit (S0033S, Beyotime Biotechnology, China). HK-2 cells were seeded in 24-well plates and cultured overnight until reaching approximately 70% confluency. To visualize mitochondria, cells were stained with MitoSO™ Red (Beyotime Biotechnology, Cat. No. S0061S) according to the manufacturer’s instructions. Following the manufacturer’s protocol for ROS detection, the cells were then stained with the specified reagents for mitochondrial ROS. Mitochondrial morphology and ROS levels were co-visualized under a fluorescence microscope (Olympus IX71, Japan).

### Target prediction of diseases and drugs

Potential targets of apigenin were identified using three bioinformatics databases: SwissTarget Prediction (http://www.swisstargetprediction), SuperPRED (https://prediction.charite.de/index.php), and PharmMapper (http://lilab-ecust.cn/pharmmapper/). Concurrently, kidney stone disease (KSD)-associated targets were retrieved from five additional databases: GeneCards (https://www.genecards.org), PharmGKB (https://www.pharmgkb.org), DrugBank (https://go.drugbank.com), OMIM (https://www.omim.org), and TTD (http://db.idrblab.net/ttd/).

### Acquisition and processing of datasets

The KSD dataset is sourced from GSE73680. This dataset aims to detect the gene expression profile of Randall plaque tissue. There is a total of 62 samples, including 6 completely normal renal papilla samples as the control group for this study, and the remaining 56 samples, including renal papilla samples with/without the presence of Randall plaques in stones (https://www.ncbi.nlm.nih.gov/).

### Differential expression analysis of KSD

The identification of differentially expressed genes was performed using Rstudio’s limma package (version 4.2.2) for differential expression analysis, with the screening criteria of logFC > 1 and *P* < 0.05 for DEGs, and visualized using volcano plots.

### Weighted gene co expression network analysis for screening potential target genes of KSD

Weighted gene co expression network analysis (WGCNA) was performed on the GSE73680 dataset using the WGCNA package in R. Sample clustering and outlier detection were conducted, and samples failing to meet the clustering threshold were excluded. Subsequently, the soft-thresholding power was tested within a range of 1 to 20, and the optimal soft threshold was determined based on both the scale-free topology fit index (*R*^2^ > 0.8) and the mean connectivity. Using the selected soft threshold, a gene co-expression adjacency matrix was constructed and transformed into a topological overlap matrix (TOM). Co-expression modules were identified using the dynamic tree cut algorithm. To merge similar modules, a merging threshold of 0.25 for module eigengenes was applied, resulting in several distinct modules. Finally, Pearson correlations between module eigengenes and sample traits were calculated to evaluate the association of each module with the disease phenotype.

### Constructing an apigenin KSD cross network and conducting GO and KEGG analysis

Analyze the target network regulated by apigenin using PPI and identify the core targets; Venn diagram reveals the intersection points of KSD and apigenin, and depicts the PPI network of intersecting targets; Key targets of apigenin therapy for KSD based on cytoHubba analysis (minimum required interaction score set to >0.4). Analyze the cross targets based on differential genes and WGCNA key modules, as well as the cross targets of apigenin KSD cross network. Perform GO and KEGG enrichment analysis using the ClusterProfiler package in R 4.2.2.

### Determination of core genes

Based on the PPI network, 15 genes were selected and further screened using three algorithms: random forest, SVM, and LASSO. The intersection of the three results was taken. Subsequently, the genes obtained from the intersection will be validated for gene expression in the dataset.

### Validation of molecular docking analysis

Crystal structure of core target from PDB database (https://www.rcsb.org/). Download the 2D structure of apigenin from PubChem database (https://pubchem.ncbi.nlm.nih.gov). Obtain and translate into 3D structure using Chemdraw software. Then use AutoDock vina to dock the target protein with apigenin.

### Cellular Thermal Shift Assay

Cellular Thermal Shift Assay (CETSA) serves as an alternative approach for evaluating drug-target protein interactions within living cells. As the temperature increases, proteins undergo degradation and show similar results. When proteins bind to drugs, the number of undecomposed proteins increases at the same temperature. Cultivate the cells until they reach a good growth state and a density of over 80%, and then extract the total protein solution. Add 8 portions of total protein solution to 150 μM API, and another 8 portions to drug solvent (DMSO) as a control. Heat the drug group and control group simultaneously at temperatures of 40, 45, 50, 55, 60, 65, 70, and 75 °C for 3 minutes, and then cool them at room temperature for 3 minutes. After the heating and cooling of all samples, centrifuge at 14000g at 4 °C for 15 minutes to remove insoluble precipitates. Take the supernatant and add 5x loading buffer in a certain proportion to denature it at high temperature. Western blot was used to detect the content of the target protein AKT1 in each group of samples.

### Drug Affinity Responsive Target Stability

Similar to the method of Zhang *et al*, extracting protein solutions from cells for Drug Affinity Responsive Target Stability (DARTS) experiments, with drug concentration set at 25, 50, 75, 100, 125, and 150 μM and enzyme concentrations of 1:1000, respectively^[^26^]^. And incubate them with different concentrations API and drug solvent (DMSO) at 37 °C for 1 hour each. After adding Pronase, lyse at room temperature for 30 minutes. Afterwards, add 5x loading buffer in a certain proportion and boil it directly. Finally, western blot was used to detect the content of the target protein AKT1 in each group of samples.

### Surface plasmon resonance

Surface plasmon resonance (SPR) was employed in this study to analyze the interaction between AKT1 protein and apigenin using a Biacore T200 system (Cytiva). AKT1 protein (100 μg) was immobilized on a CM5 sensor chip surface via amine coupling. Apigenin (5 mg) was dissolved in PBS buffer containing 1% DMSO and serially diluted into eight concentrations ranging from 0 to 100 μM. Experiments were performed in multi-cycle kinetics mode, with association and dissociation times set to 120 s and 80 s, respectively, at a flow rate of 30 μl/min. Solvent correction was carried out using a DMSO concentration series (0.8–1.2%) to subtract bulk effects. The data were fitted using both the 1:1 Langmuir binding model and the steady-state affinity model to obtain the association rate constant (ka), dissociation rate constant (kd), and equilibrium dissociation constant (KD).

### Immunofluorescence staining for nuclear translocation of Nrf2 and FOXO3 in HK-2 cells

HK-2 cells were seeded on coverslips, fixed with 4% paraformaldehyde, permeabilized with 0.1% Triton X-100, and blocked with 5% BSA. Cells were incubated overnight at 4 °C with primary antibodies against Nrf2 (Proteintech, 16396-1-AP, 1:200) and FOXO3 (Proteintech, 66428-1-Ig, 1:500). After washing, fluorescence-labeled secondary antibodies were applied for 1 hour in the dark. Nuclei were stained with DAPI. Images were acquired using a fluorescence or confocal microscope.

### Quantitative real-time PCR

Total RNA was isolated from HK-2 cells using the TRIzol method. Use TransScript one-step gDNA removal and cDNA synthesis SuperMix kit to convert extracted RNA (2 µg) into cDNA. Target gene expression was measured relative to GAPDH (endogenous control) via quantitative real-time PCR using the ABI Prism 7500 system with TransStart Green qPCR SuperMix. Primer sequences appear in Supplemental Digital Content Table S1, available at: http://links.lww.com/JS9/H40.

### Western blotting

Prepare cell lysate using RIPA buffer, collect protein samples, separate by electrophoresis, and transfer to PVDF membrane. Incubate with primary antibody, then incubate with secondary antibody, and observe protein bands using ECL assay kit. Antibody information can be found in Supplemental Digital Content Table S2, available at: http://links.lww.com/JS9/H40.

### Statistical analysis

All statistical analyses were performed using R version 4.2.2. Statistical data were analyzed with GraphPad Prism (version 8.0.2), and all values are presented as mean ± standard deviation (SD). Differences between groups were evaluated using one-way or two-way analysis of variance (ANOVA), followed by Tukey’s or Dunn’s multiple comparison post hoc test as appropriate. For comparisons between two paired groups, a paired two-tailed Student’s *t*-test was applied. A *P*-value < 0.05 was considered statistically significant, with ns indicating not significant, *P < 0.05, **P < 0.01, ***P < 0.001, and ****P < 0.0001.

## Results

### Apigenin improves renal injury induced by calcium oxalate crystals in mice

**Figure 1.**
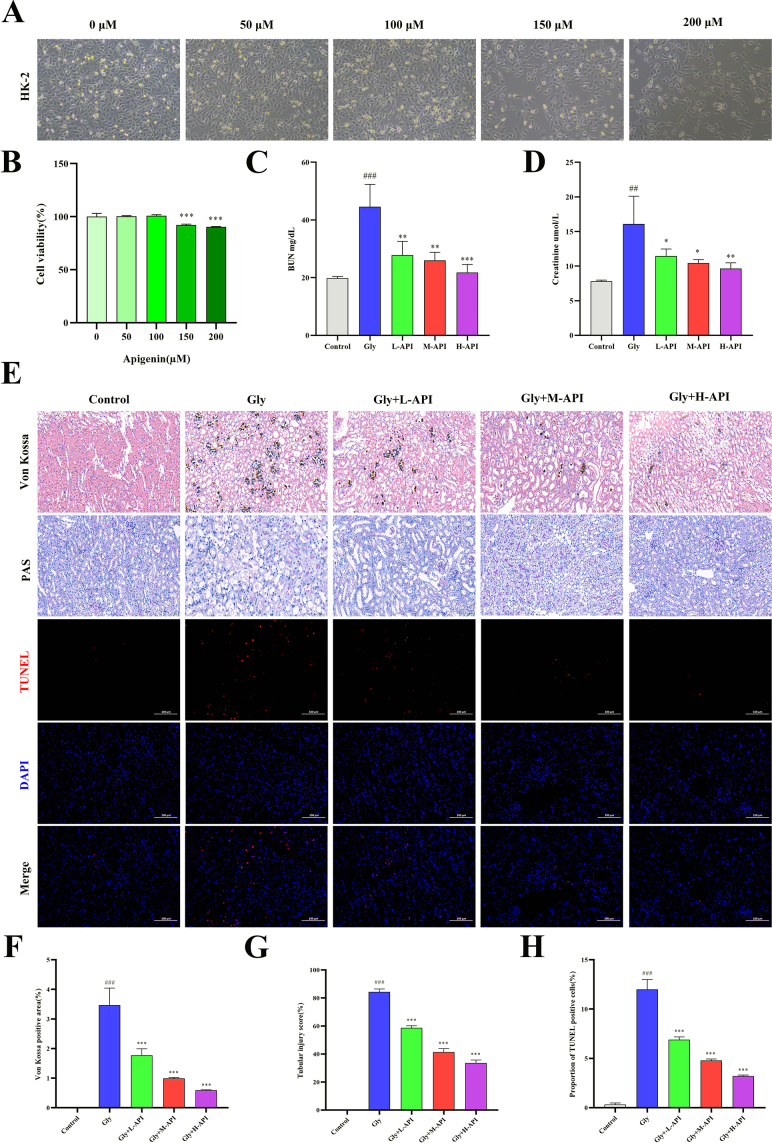
The effect of apigenin on biochemical and pathological indicators of glyoxylate – induced crystal nephropathy in mice. (A and B) The effect of different concentrations of apigenin (0, 50, 100, 150, and 200 μM) on the proliferation of HK-2 cells (*n* = 3 independent experiments). (C and D) Serum creatinine and BUN in each group (*n* = 5 independent animal samples per group). Serum creatinine and BUN levels measured in the same cohort of animals used for histological analyses. (E) PAS staining to measure tubular injury (scale bar: 50 μm); TUNEL Bright Red assay of renal apoptosis (scale bar: 50 μm); von Kossa staining shows the degree of crystal deposition in renal tissue (scale bar: 50 μm). (F–H) Quantitative analysis of von Kossa, PAS, and TUNEL staining positive areas in kidney slices. (Data are presented as mean ± SD. (*n* = 5 independent animal samples per group; ^#^*P* < 0.05 and ^##^*P* < 0.01 versus control; *P < 0.05, **P < 0.01, and ***P < 0.001 versus Gly).

We examined the effects of different concentrations of apigenin (50, 100, 150, and 200 μM) on the viability of HK-2 cells. Analysis showed that treatment for 24 hours at 100 μM had no statistically significant effect on cell proliferation, which was corroborated by morphological observations of HK-2 cells under various apigenin concentrations (Fig. 1A and B). This finding was further confirmed by cell cycle analysis via flow cytometry. The data revealed that at concentrations ≤100 μM, the proportion of cells in the DNA synthesis phase (S-phase) (~20.2–22.2%) was comparable to that of the control group (0 μM, %S = 22.2%), indicating no significant disturbance in cell cycle progression (Supplemental Digital Content Figure S3, available at: http://links.lww.com/JS9/H38). In contrast, apigenin at 150 and 200 μM significantly reduced cell viability (Fig. 1A and B). Consistently, cell cycle analysis showed a marked disruption in cell cycle distribution at these higher concentrations: the proportion of cells in G1 phase decreased significantly (to 32.7 and 36.5%, respectively), while the S-phase population increased (to 31.6 and 30.5%, respectively), suggesting possible S-phase arrest and impaired cycle completion, consistent with the observed suppression of proliferation and reduced viability (Supplemental Digital Content Figure S3, available at: http://links.lww.com/JS9/H38). Based on the integrated results of cell viability and cycle analysis, we selected 100 μM apigenin, which exhibited no significant impact on the cell cycle, for subsequent experiments.

In the animal model, we measured renal function parameters after intraperitoneal injection of glyoxylate. Compared with the control group, apigenin treatment ameliorated the decline in renal function (Fig. 1C and D). Consistent with the biochemical indices, pathological examination revealed calcium oxalate crystal deposition in the renal parenchyma of the glyoxylate group. Mice treated with apigenin showed a significant, dose-dependent reduction in renal CaOx crystal deposition, lessened tubular epithelial injury, and decreased numbers of dead tubular epithelial cells (Fig. 1E**–**H).

### Pharmacokinetic profile of apigenin in plasma and kidney tissue

To evaluate the bioavailability and tissue distribution of apigenin, we measured its concentration in plasma and kidney tissues at various time points after oral administration. The results are summarized in Supplemental Digital Content Tables S3 and S4, available at: http://links.lww.com/JS9/H40. In plasma, apigenin was rapidly absorbed, reaching peak concentrations (*C_max_*) at 2 hours post-administration. The mean *Cmax* values were 309.32 ng/ml, 848.61 ng/ml, and 1242.07 ng/ml for the low-, medium-, and high-dose groups, respectively. Apigenin was detectable up to 8 hours, indicating sustained systemic exposure. In kidney tissues, apigenin accumulation was dose-dependent and time-dependent. The highest concentrations were observed at 2 hours, with mean values of 692.35, 1771.23, and 2528.88 ng/g for the three dose groups. Notably, apigenin levels in kidney tissues remained elevated at 8 hours, suggesting effective tissue penetration and retention. These findings demonstrate that orally administered apigenin is efficiently absorbed and accumulates in renal tissues, supporting its potential as a therapeutic agent for kidney stone disease.

### Identification of differentially expressed genes in KSD patients and construction of WGCNA

**Figure 2.**
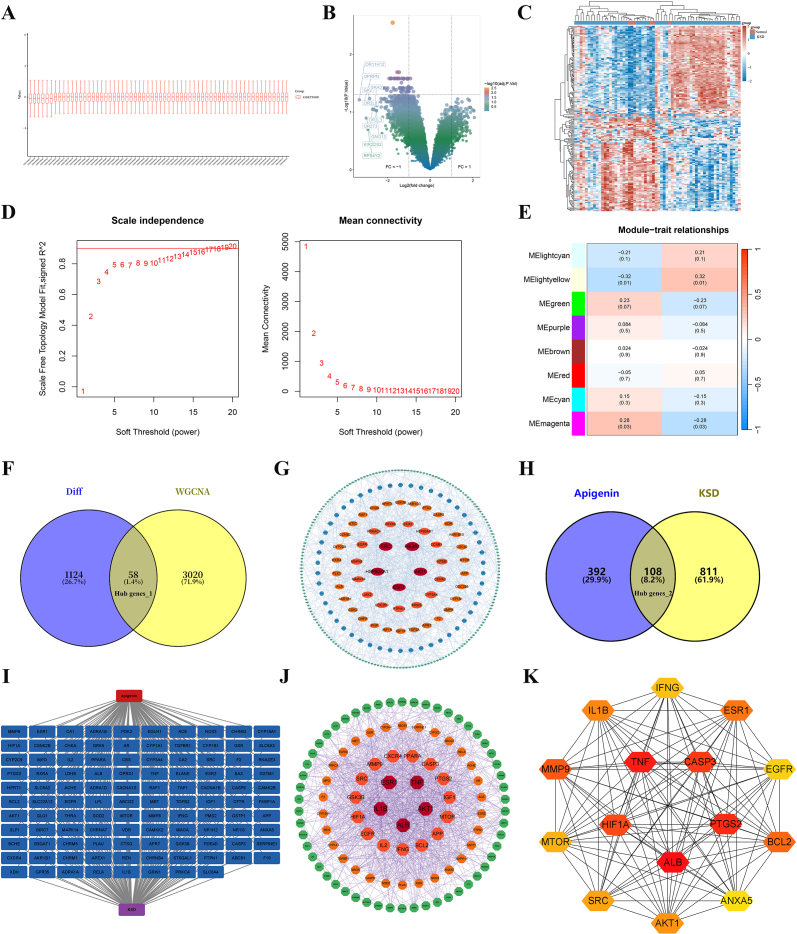
Integrated network pharmacology and bioinformatics analysis identifies potential therapeutic targets of apigenin in kidney stone disease. (A) Standardization of RNA microarray data in GSE73680 dataset. (B) Volcano plots of DEG distributions contained within the GSE73680 dataset. (C) Heatmap displaying differential gene distribution in KSD and normal patients. (D) Analysis of the scale-free fit index (left) and the mean connectivity (right) for various soft-thresholding power value. (E) Heatmap showing the correlation between modules and feature gene sets. (F) Select overlapping genes between DEGs and key module genes related to KSD. (G) PPI target network regulated by apigenin target. (H and I) 108 cross indicators of apigenin and KSD (hub genes_2). (J) PPI network of apigenin and KSD cross indicators. (K) Key indicators for apigenin treatment of KSD based on cytoHubba analysis.

According to our processing of the GSE73680 dataset, RNA microarray data was standardized and analyzed for differences (Fig. 2A), and final screening was performed (log | FC | > 1, *P* < 0.05) to obtain 1182 differentially expressed genes, of which 205 were up-regulated and 977 were down-regulated (Fig. 2B and C). A weighted gene co-expression network was constructed using WGCNA. Sample clustering based on Pearson correlation coefficients was performed to generate a dendrogram, followed by selection of the optimal soft-thresholding power (β = 5, R^2^ = 0.8) to establish a scale-free network topology (Fig. 2D). Following adjacency matrix construction, a topological overlap matrix (TOM) was generated. Module-trait correlation analysis revealed that the lightyellow module exhibited the strongest positive association with kidney stone disease status (cor = 0.32, *P* = 0.01; Fig. 2E). To identify functionally relevant candidates, the 3,078 genes within the lightyellow module were intersected with the 1,182 differentially expressed genes (|logFC| > 1, *P* < 0.05), yielding 58 overlapping genes (Hub gene_1; Fig. 2F), which were subsequently subjected to GO and KEGG enrichment analyses.

### The construction of target and intersecting target network for apigenin and KSD

919 KSD targets were obtained from DrugBank, GeneCards, OMIM, TTD, and PharmGKB databases; A total of 500 apigenin targets were obtained from Pharmmapper, Swiss Target Prediction, and Super PRED databases. A total of 440 nodes and 780 edges were found in the PPI network through the construction of the apigenin target network (Fig. 2G). After overlapping apigenin KSD targets, 108 common targets were screened (Fig. 2H, Hub gene_2), and the apigenin-KSD-target network diagram was utilized using Cytoscape software (Fig. 2I). Based on cytoHubba analysis, a total of 107 nodes and 1106 edges were identified. Based on MCC analysis, these targets were screened separately, therefore the hub genes for apigenin treatment of KSD are *TNF, CASP3, ALB, PTGS2, MMP9, BCL2, HIF1A, ESR1, IL1B, AKT1, SRC, MTOR, IFNG, EGFR*, and *ANXA5* (Fig. 2J and K).

### GO and KEGG analysis based on two different screening methods for potential targets

**Figure 3.**
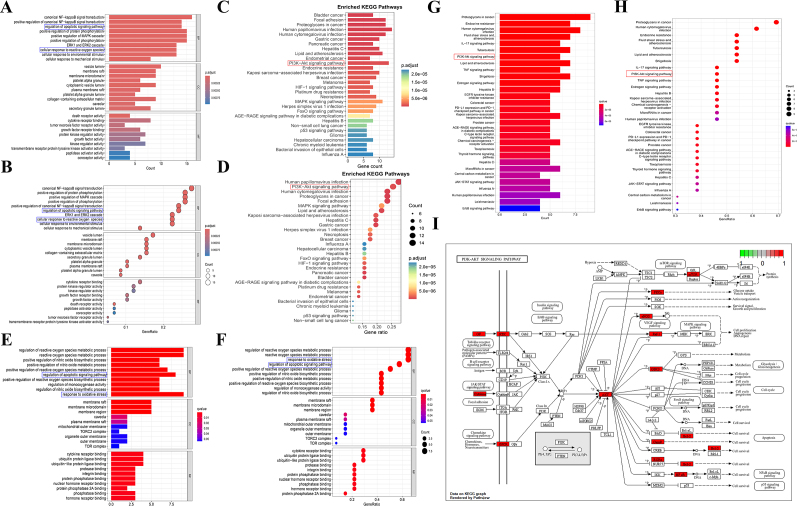
Biological functional analysis of hub genes_1 and hub genes_2. (A and B) GO analysis of hub genes_1. (C and D) KEGG analysis of hub genes_1. (E and F) GO analysis of hub genes_2. (G and H) KEGG analysis of hub genes_2. (I) Detailed information on the enrichment of key genes in the PI 3K/AKT signaling pathway.

To determine the key pathways involved in apigenin in KSD, we performed GO and KEGG analyses on Hub gene_1 and Hub gene_2, respectively. The GO analysis based on Hub gene_1 showed that the results indicated the top 10 biological processes, cellular components, and molecular functions in the enriched entries. Our analysis shows that genes associated with these enriched GO terms are mainly involved regulation of apoptotic signaling pathway, collagen fibril organization, cartilage development and the regulation of apoptotic signaling pathway (Fig. 3A and B). In addition, in the KEGG analysis results, the 16 most significantly enriched pathways were enriched, among which Focal adhesion, Human papillomavirus infection、 ECM receptor interaction and PI3K-Akt signaling pathway rank among the top few (Fig. 3C and D).

In addition, the GO analysis results of Hub gene_2 showed that its genes are mainly involved in reactive oxygen species metabolism, oxidative stress response, and apoptosis signaling pathways (Fig. 3E and F). In addition, in the results of KEGG analysis, the 30 most significantly enriched pathways were enriched, among which the pathways that best matched the research objectives included IL-17 signaling pathway, HIF-1 signaling pathway, and TNF signaling pathway (Fig. 3G and H). Overall, we can be surprised to find from the enrichment analysis of Hub gene_1 and Hub gene_2 that the genes share similarities in biological processes, cellular components, molecular functions, or pathways. Therefore, we speculate that apigenin may affect KSD through the PI3K-AKT signaling pathway. However, when mapping common targets to the AKT signaling pathway and considering the enrichment of the signaling pathway, PI3K was not enriched; Therefore, we hypothesize that apigenin may directly act on AKT1, regulate its downstream, and thus affect KSD (Fig. 3I).

### Determination of the core gene AKT1

**Figure 4.**
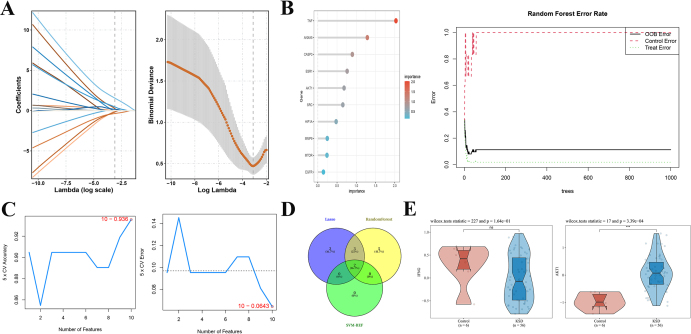
Using machine learning combined with network pharmacology results to screen AKT1. (A) LASSO model’s cross-validation process for adjusting parameter selection. Each curve represents a single gene. LASSO analysis of the coefficients. Plotted at the best lambda are vertical dashed lines. (B) The correlation between the total number of trees in the random forest and the error rates. An order based on the relative significance of the genes. (C) SVM-RFE approach for the selection of feature genes. (D) Intersection results of three algorithms. (E) Expression of AKT1 and IFNG in KSD and normal groups. *P < 0.05, **P < 0.01, and ***P < 0.001.

Based on 15 PPI potential genes, LASSO, SVM, and random forest algorithms were used for core gene screening. The lasso model obtained seven genes (Fig. 4A). Select the top 10 feature genes in the random forest algorithm (Fig. 4B). The results of the SVM algorithm show that two genes were included in the ranking based on AvgRank combined with inflection points (Fig. 4C). Then we crossed them to obtain IFNG and AKT1 (Fig. 4D). Finally, observe the expression of two genes in the disease and normal groups in the database. It was found that only the expression of AKT1 differed between the normal group and the disease group, and its expression level was higher in patients with stones (Fig. 4E). Therefore, based on the above results, we have decided to focus our research on AKT1.

### Apigenin directly targets AKT1 and specifically regulates it signaling pathway

**Figure 5.**
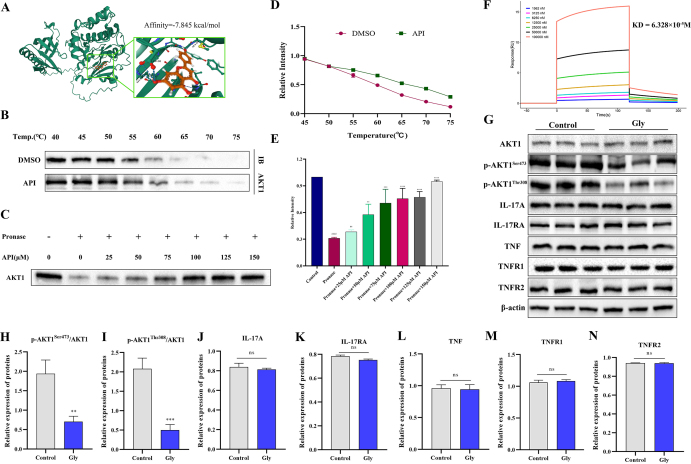
Validation of the targeted binding relationship between apigenin and AKT1. (A) Molecular docking of apigenin complexed with AKT1. (B and D) CETSA – Immunoblotting showed an increase in thermal stability of the target protein AKT1 after binding to apigenin (*n* = 3 independent experiments). (C and E) DARTS immunoblotting showed an increase in protease stability after binding the target protein AKT1 to apigenin (*n* = 3 independent experiments). (F) SPR analysis confirming high-affinity interaction between apigenin and AKT1. (G–N) Western blot analysis of key signaling pathway proteins to confirm the specificity of AKT1 activation by apigenin in a glyoxylate-induced renal injury model. Phosphorylation levels of AKT1 (Ser473 and Thr308) and expression of IL-17/TNF-α pathway proteins were assessed (*n* = 3 independent animal samples per group). Data are presented as mean ± SD. ^#^*P* < 0.05, ^##^*P* < 0.01 vs Control; **P < 0.01, ***P < 0.001 vs Gly; “ns” indicates not significant.

To confirm that API exerts its renoprotective effects via AKT1, a series of experimental validations were performed. To verify binding specificity, molecular docking simulations revealed that apigenin stably occupies the active pocket of AKT1, with a binding energy of −7.845 kcal/mol (Fig. 5A). CETSA and quantitative analysis demonstrated that apigenin treatment significantly enhanced the thermal stability of AKT1 compared to the DMSO control (Fig. 5B and D). In DARTS assays conducted with a concentration gradient, increasing apigenin concentrations progressively strengthened the resistance of AKT1 to proteolytic digestion, indicating a clear dose-dependent protective effect (Fig. 5C and E). Furthermore, SPR confirmed a high-affinity interaction between apigenin and AKT1 protein, with an equilibrium dissociation constant (KD) of 6.328 × 10^−^⁵ M (Fig. 5F).

To elucidate the activation mechanism of AKT1, its key phosphorylation sites were examined. Western blot analysis showed that apigenin treatment markedly increased the phosphorylation levels of AKT1 at both critical sites, Ser473 and Thr308 (Fig. 5G**–**I). Notably, the levels of upstream regulators PI3K p85 and PDK1 did not differ significantly between the model and control groups, ruling out the possibility that apigenin indirectly activates AKT1 via upstream kinases and supporting a direct activation mechanism.

To further verify pathway specificity, other potential signaling pathways were systematically evaluated. Results indicated that in the glyoxylate-induced renal injury model, key proteins of the IL-17 signaling pathway and molecules associated with the TNF-α pathway (TNF-α, TNFR1, TNFR2) exhibited no significant alterations (Fig. 5G and J–N). Additionally, cleaved-caspase-8, a key marker of death receptor-mediated extrinsic apoptosis, showed no notable difference between the model and control groups (Supplemental Digital Content Figure S4, available at: http://links.lww.com/JS9/H39), excluding a major contribution of this pathway. Together, these findings demonstrate that apigenin directly and specifically binds to AKT1, activates its dual-site phosphorylation, and exerts its core protective action predominantly through the AKT1/mTOR pathway, rather than via the upstream PI3K/PDK1 axis, IL-17/TNF signaling, or death receptor-mediated apoptotic routes.

### Apigenin can inhibit cell apoptosis induced by calcium oxalate crystals and enhance antioxidant capacity

**Figure 6.**
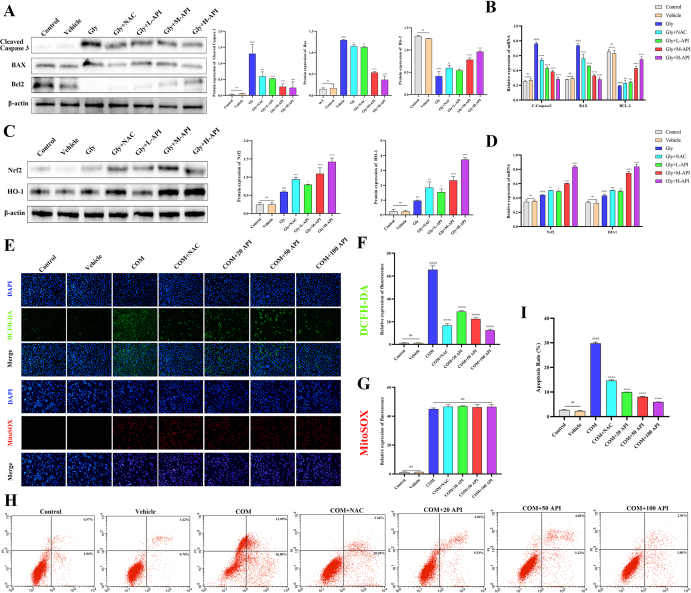
Regulation of apigenin on renal cell apoptosis and oxidative stress induced by calcium oxalate crystals. (A and B) The protein and mRNA expression of apoptosis related proteins BAX, BCL2, and caspase-3 in renal tissue from Control, Vehicle, Gly, Gly + L-API, Gly + M-API, Gly + H-API, and Gly + NAC groups. Quantification of protein levels normalized to β-actin (*n* = 3 independent animal samples per group). (C and D) The protein and mRNA expression of the antioxidant proteins Nrf2 and HO-1 in kidney tissues from Control, Vehicle, Gly, Gly + L-API, Gly + M-API, Gly + H-API, and Gly + NAC groups (*n* = 3 independent animal samples per group). (E and F) Immunofluorescence detection of total intracellular ROS levels in HK-2 cells from Control, COM, Vehicle, COM + L-API, COM + M-API, COM + H-API, and COM + NAC groups (*n* = 3 independent experiments). Scale bar: 50 μm. (E and G) MitoSOX staining for mitochondrial ROS in HK-2 cells from the same treatment groups (*n* = 3 independent experiments). (H and I) Flow cytometry analysis of apoptosis in HK-2 cells from Control, Vehicle, COM, COM + L-API, COM + M-API, COM + H-API, and COM + NAC groups (*n* = 3 independent experiments). All data are presented as mean ± SD. ^##^*P* < 0.01, ^###^*P* < 0.001, ^####^*P* < 0.0001 vs Control; *P < 0.05, **P < 0.01, ***P < 0.001, ****P < 0.0001 vs Gly; “ns” indicates not significant.

To elucidate the downstream biological effects resulting from the direct binding and activation of AKT1 by API, we further evaluated its anti-apoptotic and antioxidant functions. TUNEL staining indicated apoptosis induction in the Gly model, and subsequent protein and gene-level analyses confirmed that the Gly group exhibited significantly elevated expression of the pro-apoptotic proteins BAX and cleaved-caspase-3, along with reduced expression of the anti-apoptotic protein BCL2. API intervention dose-dependently reversed these alterations, with effects superior to those of the positive control NAC (Fig. 6A and B). Regarding oxidative stress, expression of the key antioxidant factor Nrf2 and its downstream target HO-1 was decreased in the Gly group, and both apigenin and NAC treatments effectively restored their expression (Fig. 6C and D). Immunofluorescence detection revealed that apigenin markedly suppressed total intracellular ROS levels (Fig. 6E and F), while MitoSOX staining indicated no significant change in mitochondrial ROS (Fig. 6G), suggesting that the antioxidant effect of apigenin may primarily stem from scavenging cytosolic free radicals rather than targeting mitochondrial oxidative stress. Furthermore, flow cytometry corroborated that apigenin attenuated Gly-induced apoptosis (Fig. 6H and I). Combined with earlier results showing no significant alteration in the key extrinsic apoptosis molecule cleaved-caspase-8 (Supplemental Digital Content Figure S4, available at: http://links.lww.com/JS9/H39), these data imply that the anti-apoptotic action of apigenin likely operates predominantly through modulation of the mitochondrial pathway. In summary, apigenin, via direct binding to AKT1 and activation of its downstream signaling, exerts dose-dependent anti-apoptotic and antioxidant effects that surpass those of NAC.

### Apigenin regulates cell apoptosis and oxidative stress through the AKT1/mTOR and AKT1/FOXO3 pathways

**Figure 7.**
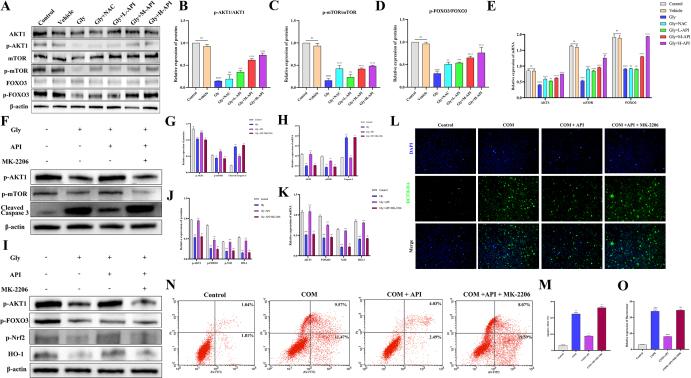
The effects of different concentrations of apigenin on the AKT1/mTOR and AKT1/FOXO3 pathways. (A–D) Western blot analysis of phosphorylated and total protein levels of AKT1, mTOR, and FOXO3 in kidney tissues from Control, Vehicle, Gly, Gly + L-API, Gly + M-API, and Gly + H-API groups (*n* = 3 independent animal samples per group). (E) Quantitative PCR analysis of AKT1, mTOR, and FOXO3 mRNA levels in kidney tissues from the same groups (*n* = 3 independent animal samples per group). (F–H) Western blot and qPCR analysis of AKT1, mTOR, and cleaved-caspase-3 expression in kidney tissues from Control, Vehicle, Gly, Gly + H-API, and Gly + H-API + MK-2206 groups after treatment with the AKT1 inhibitor MK-2206 (*n* = 3 independent animal samples per group). (I–K) Western blot and qPCR analysis of AKT1, FOXO3, Nrf2, and HO-1 expression in kidney tissues from the same groups (*n* = 3 independent animal samples per group). (L and O) Immunofluorescence detection of intracellular ROS levels in HK-2 cells from Control, COM, COM + API, and COM + API + MK-2206 groups (n = 3 independent experiments). Scale bar: 50 μm. (N and M) Flow cytometry analysis of apoptosis in HK-2 cells from the same treatment groups (n = 3 independent experiments). All data are presented as mean ± SD. ^#^*P* < 0.05 and ^##^*P* < 0.01 versus control; *P < 0.05 and **P < 0.01, versus Gly; “ns” indicates no significant *P < 0.05; **P <0.01, ***P < 0.001; ****P < 0.0001.

These results indicate that API effectively suppresses calcium oxalate crystal-induced oxidative stress and apoptosis. To clarify the specific molecular pathways through which it alleviates renal injury, we further investigated the downstream signaling mechanisms by which API modulates these two pathological phenotypes. Prior literature has established that both mTOR and FOXO3 function as key downstream effectors of AKT1^[^27,28^]^. The mTOR pathway is involved in the regulation of apoptosis^[^29^]^. Conversely, FOXO3 modulates oxidative stress via the Nrf2/HO-1 pathway^[^30,31^]^. Consequently, we postulated that API might confer its protective effects through modulation of two parallel pathways: AKT1/mTOR and AKT1/FOXO3. Quantitative analysis via qPCR and Western blotting revealed that API administration significantly and dose-dependently elevated both protein and mRNA levels of p-AKT1/AKT1, p-mTOR/mTOR, and p-FOXO3/FOXO3 in mouse kidney tissues (Fig. 7A–E). These findings indicate that API activates AKT1 signaling *in vivo*, subsequently stimulating the downstream mTOR pathway and inducing FOXO3 phosphorylation, a hallmark of suppressed FOXO3 transcriptional activity.

To establish the necessity of these pathways in API-mediated protection, we first employed the AKT1-specific inhibitor MK-2206. Western blot and qPCR analyses demonstrated that MK-2206 effectively suppressed the API-induced upregulation of AKT1 and mTOR in the Gly-induced renal injury model. Concurrently, MK-2206 treatment markedly enhanced the expression of Cleaved Caspase-3, indicating reactivation of the apoptotic cascade (Fig. 7F–H). Regarding oxidative stress, MK-2206 similarly abrogated the protective effect of API, significantly downregulating the expression of AKT1, FOXO3, Nrf2, and HO-1 (Fig. 7I–K). Further *in vitro* validation confirmed these observations: ROS fluorescence assays showed that MK-2206 counteracted the inhibitory effect of API on COM crystal-induced oxidative stress (Fig. 7L and M), while flow cytometric apoptosis analysis revealed that MK-2206 treatment eliminated the protective effect of API against COM crystal-induced apoptosis (Fig. 7N and O). Collectively, these results indicate that both the AKT1/mTOR and AKT1/FOXO3 pathways are critically involved in the anti-apoptotic and antioxidant effects mediated by API.

### AKT1 knockdown attenuates API-mediated anti-apoptotic and antioxidant effects

**Figure 8.**
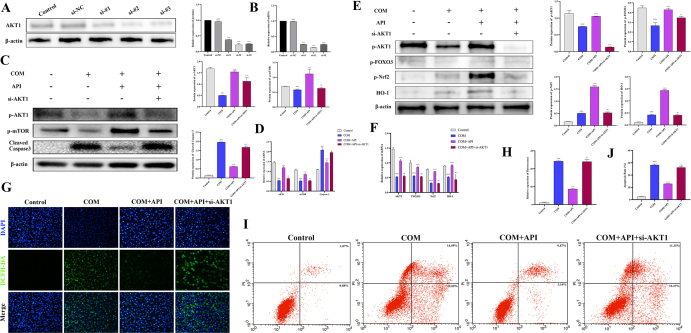
AKT1 knockdown attenuates apigenin-mediated anti-apoptotic and antioxidant effects *in vitro*. (A and B) Western blot and qPCR confirmation of AKT1 knockdown efficiency in HK-2 cells transfected with control siRNA (si-NC) or AKT1-specific siRNA (si-AKT1) (*n* = 3 independent experiments). (C and D) The protein and mRNA expression of AKT1, mTOR and cleaved-caspase-3 in HK-2 cells from Control, COM, COM + API, COM + API + si-AKT1 groups (*n* = 3 independent experiments). (E and F) The protein and mRNA expression of AKT1, FOXO3, Nrf2, and HO-1 in HK-2 cells (*n* = 3 independent experiments). (G and H) Immunofluorescence detection of intracellular ROS levels in HK-2 cells from Control, COM, COM + API, COM + API + si-AKT1 groups (*n* = 3 independent experiments). Scale bar: 50 μm. (I, J) Flow cytometry analysis of apoptosis in HK-2 cells from the same treatment groups (*n* = 3 independent experiments). All data are presented as mean ± SD. ^#^*P* < 0.05 and ^##^*P* < 0.01 versus control; *P < 0.05 and **P < 0.01, versus Gly; “ns” indicates no significant *P < 0.05; **P <0.01; ***P < 0.001.

To further validate the functional necessity and specificity of AKT1 at the genetic level, we performed siRNA-mediated knockdown of AKT1. Western blot and qPCR confirmed that si-AKT1 effectively reduced both protein and mRNA levels of AKT1 in HK-2 cells (Fig. 8A and B). Under AKT1-knockdown conditions, the API-induced upregulation of p-mTOR and p-FOXO3 was significantly suppressed (Fig. 8C and E), indicating that activation of these downstream pathways strictly depends on API-mediated regulation of AKT1. Moreover, AKT1 knockdown attenuated the anti-apoptotic effect of API, as evidenced by a diminished inhibition of Cleaved Caspase-3 expression (Fig. 8C and D), and an overall reduction in the anti-apoptotic efficacy of API (Fig. 8I and J). Regarding oxidative stress, si-AKT1 also blocked the antioxidant action of API, abolishing its promotive effects on Nrf2 and HO-1 expression (Fig. 8E and F) and weakening its suppression of intracellular ROS accumulation (Fig. 8G and H). Together, these data demonstrate that AKT1 serves as an essential molecular hub through which API orchestrates downstream mTOR and FOXO3 signaling to exert anti-apoptotic and antioxidant activities. Further support the core position of AKT1 in this protection mechanism.

### Apigenin activates the Nrf2 antioxidant pathway via AKT1-mediated FOXO3 inhibition and subsequent Keap1 downregulation

**Figure 9.**
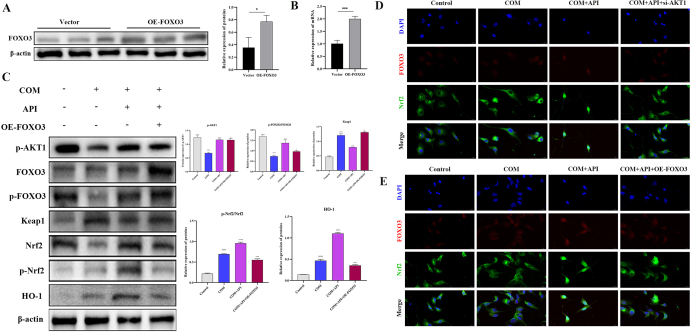
Apigenin activates the Nrf2 antioxidant pathway via AKT1-mediated FOXO3 inhibition and subsequent Keap1 downregulation. (A and B) Western blot and qPCR confirmation of efficient FOXO3 overexpression (OE-FOXO3) in HK-2 cells (*n* = 3 independent experiments). (C) The protein and mRNA expression of AKT1, FOXO3, Keap1, Nrf2, and HO-1 protein expression in HK-2 cells from Control, COM, COM + API, and COM + API + OE-FOXO3 groups (*n* = 3 independent experiments). (D) Immunofluorescence analysis of Nrf2 (green) and FOXO3 (red) subcellular localization in HK-2 cells from Control, COM, COM + API, and COM + API + si-AKT1 groups (*n* = 3 independent experiments). Scale bar: 20 μm. (E) Immunofluorescence analysis of Nrf2 (green) and FOXO3 (red) subcellular localization in HK-2 cells from Control, COM, COM + API, and COM + API + OE-FOXO3 groups (*n* = 3 independent experiments). Scale bar: 20 μm. All data are presented as mean ± SD. ^#^*P* < 0.05, ^##^*P* < 0.01 vs Control; *P < 0.05, **P < 0.01, ***P < 0.001 vs COM.

Collectively, both genetic and pharmacological evidence confirms that AKT1 activation is a prerequisite for API-mediated renal protection, with its downstream signaling encompassing both mTOR and FOXO3. Notably, although FOXO3 transcriptional activity was suppressed, API treatment concurrently and significantly upregulated the expression of the core antioxidant factor Nrf2 and its downstream target HO-1. This observation diverges from the classical paradigm in which FOXO3 can directly transactivate antioxidant genes under certain conditions. This discrepancy suggests that in the specific pathological context of calcium oxalate crystal-induced injury, FOXO3 may play a non-canonical role, potentially via an API-triggered “AKT1–FOXO3–Keap1–Nrf2” regulatory axis. Recent studies have highlighted the context-dependent functionality of FOXO3. For instance, in cholangiocarcinoma cells, FOXO3 acts as a key transcriptional activator of the Keap1 gene; its inactivation reduces Keap1 expression, thereby alleviating Keap1-mediated degradation of Nrf2 and ultimately activating the Nrf2 pathway to enhance cellular antioxidant capacity^[^32^]^. Inspired by this finding, we performed gain-of-function experiments to investigate whether FOXO3 mediates the antioxidant effect of API by regulating Keap1. Overexpression of FOXO3 (OE-FOXO3) in HK-2 cells significantly elevated both FOXO3 protein and mRNA levels, as confirmed by Western blotting and qPCR (Fig. 9A and B). OE-FOXO3 markedly blocked API-induced downregulation of Keap1, restoring Keap1 protein to a higher level. Importantly, FOXO3 overexpression did not alter API-induced AKT1 activation, as p-AKT1 levels remained unchanged, indicating that FOXO3 acts downstream of AKT1 to regulate Keap1 expression. Consequently, the API-induced nuclear accumulation of Nrf2 and upregulation of HO-1 protein expression were also substantially reversed (Fig. 9C). To further elucidate the molecular events underlying AKT1-mediated regulation of FOXO3 and its functional association with the antioxidant phenotype, we assessed the nuclear translocation of FOXO3 (red) and Nrf2 (green). Immunofluorescence analysis indicated that API treatment promoted nuclear accumulation of Nrf2 and enhanced cytoplasmic translocation of FOXO3 (Fig. 9D and E). Importantly, siRNA-mediated AKT1 knockdown reversed this pattern, resulting in FOXO3 relocalization to the nucleus and abrogation of Nrf2 nuclear accumulation (Fig. 9D). Consistent with these observations, FOXO3 overexpression also suppressed API-induced nuclear translocation of Nrf2 (Fig. 9E). Together, these findings demonstrate that in calcium oxalate crystal-induced renal injury, API activates AKT1, leading to phosphorylation-dependent inhibition of nuclear FOXO3 function. This downregulates the expression of its target gene Keap1, thereby relieving Keap1-mediated repression and activating the Nrf2-driven antioxidant response. This work reveals a non-canonical pathway distinct from the classical model in which FOXO3 directly transactivates antioxidant genes, highlighting the context-dependent functionality of FOXO3 under renal pathological stress.

## Discussion

Renal calculi represent a significant global health burden, contributing to substantial morbidity, impaired quality of life, and considerable healthcare expenditures^[^33^]^. The recurrent nature of this condition poses particular challenges, with reported recurrence rates ranging from 30% to 80% within a decade following initial diagnosis^[^34^]^. This high propensity for stone reformation necessitates ongoing clinical management and underscores the importance of effective preventive strategies. Mechanistically, CaOx crystals exhibit significant cytotoxicity towards renal tubular epithelial cells, leading to cell death and crystal deposition, ultimately resulting in renal calcium deposition and even renal failure^[^35^]^.

Although studies have shown that apigenin has a protective effect on kidney injury in a rat model of urolithiasis, the exact *in vitro* and *in vivo* anti apoptotic, antioxidant functions, and potential specific molecular mechanisms of apigenin are still unclear and require further research^[^20^]^. Here, this study suggests that apigenin can alleviate kidney damage induced by calcium oxalate crystals. In terms of mechanism, we first discovered that apigenin can target AKT1 and activate the phosphorylation of AKT1 to have downstream effects, thereby preventing cell apoptosis and ROS production and exerting antioxidant effects. The research results revealed a new molecular mechanism by which apigenin exerts its anti-oxalate calcium crystal induced renal injury effect.

We initially investigated the effect of API on calcium oxalate crystal-induced kidney injury and conducted toxicological evaluations, preliminarily confirming its safety profile. Pharmacokinetic analyses demonstrated that orally administered API is effectively absorbed and accumulates in renal tissues in a dose-dependent manner, providing pharmacokinetic support for its local protective action in the kidneys. In functional experiments, API at doses of 10, 25, and 50 mg/kg exhibited significant renal protective effects in a dose-dependent manner. By integrating WGCNA, machine learning, and network pharmacology, we identified AKT1 and IFNG as potential key therapeutic targets of API for KSD. Further bioinformatic analysis of human KSD transcriptomic data (GSE73680) revealed significant alterations in AKT1 expression, whereas IFNG and other candidates did not show consistent changes. KEGG and GO enrichment analyses further indicated significant enrichment of pathways related to “PI3K-AKT signaling,” “apoptosis,” and “oxidative stress response” under disease conditions. Notably, although components of inflammatory pathways such as TNF and IL-17 were also modestly enriched in human datasets, our *in vivo* experiments did not detect significant changes in key proteins of these pathways. This suggests that in the acute crystal-induced injury model employed in this study, the protective effects of API are likely mediated through direct regulation of AKT1, thereby influencing apoptosis and oxidative stress pathways, rather than via modulation of the aforementioned inflammatory pathways. Therefore, we hypothesize that API may exert therapeutic effects on KSD by targeting AKT1 to modulate cellular apoptosis and oxidative stress.

The mTOR and FOXO signaling pathways serve as crucial downstream effectors of Akt, each exhibiting distinct regulatory mechanisms in biological systems^[^36–40^]^. The mTOR activation has been shown to inhibit apoptotic cascades through modulation of mitochondrial outer membrane permeability^[^41^]^. This process involves ROS-mediated alterations that trigger the release of pro-/anti-apoptotic factors from the Bcl-2 protein family, ultimately culminating in caspase-3 activation and programmed cell death^[^42^]^. Among the FOXO family members, FOXO3 demonstrates predominant expression in renal tissues and plays a pivotal role in oxidative stress regulation^[^43^]^. In calcium oxalate crystal-induced nephropathy, excessive ROS accumulation disrupts cellular homeostasis, leading to oxidative damage and mitochondrial dysfunction^[^44,45^]^. The Akt/Nrf2/HO-1 axis represents a well-characterized pathway for oxidative stress modulation, primarily through enhancement of cellular antioxidant capacity^[^46,47^]^. Emerging evidence suggests that FOXO3 may exert its protective effects via interaction with the Nrf2/HO-1 signaling cascade, highlighting its potential as a therapeutic target in oxidative injury^[^48,49^]^. The experimental results demonstrated that apigenin activates the phosphorylation of AKT1, leading to subsequent alterations in the downstream AKT1/mTOR and AKT1/FOXO3 signaling pathways. Consequently, increased mTOR phosphorylation suppresses the production of the pro-apoptotic protein BAX and promotes the expression of the anti-apoptotic protein BCL-2. Meanwhile, enhanced phosphorylation of FOXO3 initiates critical subcellular localization changes. This study revealed that apigenin treatment promotes AKT1-mediated phosphorylation of FOXO3, resulting in the translocation of FOXO3 from the nucleus to the cytoplasm, thereby inhibiting its transcriptional activity. Correspondingly, a significant enhancement in Nrf2 nuclear translocation was observed. Collectively, these changes downregulate the expression of the target gene Keap1, which relieves Keap1-mediated degradation of Nrf2, facilitates Nrf2 nuclear translocation, and upregulates the transcription of its downstream target gene HO-1, ultimately augmenting cellular antioxidant capacity to scavenge reactive oxygen species. Nrf2 is an important transcription factor that contributes to the expression of antioxidant defense genes. Under normal conditions, the transcriptional activity of Nrf2 is blocked^[^50^]^. Once activated, Nrf2 translocates to follow oxidative stress induction by ROS, Nrf2 translocates to the nucleus and interacts with the antioxidant response element (ARE) – a cis-regulatory sequence located in antioxidant gene promoters^[^51^]^. This evolutionarily conserved Nrf2-ARE signaling cascade serves as a critical defense mechanism, safeguarding cellular homeostasis against oxidative damage^[^52^]^. Therefore, in the context of calcium oxalate crystal-induced injury, apigenin activates AKT1, leading to the phosphorylation and subsequent nuclear export of FOXO3, thereby inactivating it. Concurrently, this pathway indirectly promotes the nuclear translocation and activation of Nrf2, establishing a coordinated transcriptional regulatory network. Additionally, recognizing the close interplay between ROS and apoptosis, we further investigated whether the anti-apoptotic effect of apigenin is primarily a direct consequence of AKT1/mTOR activation or a secondary result of mitigated oxidative stress. In a COM-induced HK-2 cell model, using the potent antioxidant N-acetylcysteine (NAC), we observed that although NAC effectively reduced intracellular ROS, it only partially attenuated apoptosis. In contrast, apigenin provided significantly stronger protection. This observation aligns with the well-established intimate and often interdependent relationship between ROS production and apoptosis initiation, as reported in numerous studies^[^53–55^]^. This indicates that the anti-apoptotic effect of apigenin is partly attributable to its antioxidant capacity in alleviating ROS-induced damage but is predominantly driven by direct activation of the AKT1/mTOR pathway, establishing a synergistic network wherein the reduction of oxidative stress may further mitigate ROS-triggered apoptotic signaling.

Mechanistically, MK-2206, an allosteric AKT inhibitor, suppresses AKT signaling by preventing subcellular translocation and inhibiting phosphorylation-dependent kinase activation^[^56^]^. In renal cells exposed to calcium oxalate crystals, pharmacological inhibition with MK-2206 effectively abolished apigenin-mediated regulation of the AKT1/mTOR and AKT1/FOXO3 signaling cascades, thereby counteracting the therapeutic effects of apigenin in both *in vitro* and *in vivo* models. To further confirm the specificity of this action, siRNA-mediated knockdown of AKT1 similarly blocked the activation of downstream signaling pathways and the protective effects of apigenin. Conversely, overexpression of FOXO3 reversed apigenin-induced downregulation of Keap1 and activation of Nrf2. These results collectively demonstrate that the anti-apoptotic and antioxidant effects of apigenin against nephrolithiasis are primarily mediated through the specific modulation of AKT1-dependent pathways. The apigenin-AKT1 axis identified in this study provides a novel strategic approach for the prevention and treatment of kidney stones. From a surgical perspective, current postoperative prevention for recurrent kidney stone patients primarily relies on lifestyle modifications and pharmacological interventions (e.g., citrate, thiazide diuretics), yet some patients still face challenges with recurrence or drug intolerance. As a natural flavonoid compound with multi-target and low-toxicity characteristics, apigenin may serve as an effective complement to existing preventive regimens.

Although the glyoxylate injection model used in this study effectively and rapidly simulates acute calcium oxalate crystal deposition and tubular injury, facilitating the evaluation of apigenin’s acute protective effects against oxidative stress and apoptosis, it differs from human kidney stone disease in certain aspects^[^4,5^]^. Human nephrolithiasis often manifests as a chronic and recurrent condition, involving complex pathological processes such as persistent crystal deposition, interstitial inflammation, fibrosis, and Randall’s plaque formation^[^57^]^. Therefore, the findings of this study primarily elucidate the role of apigenin in alleviating initial crystal-induced renal injury. To translate these findings from basic research to clinical application, future studies should focus on the following systematic investigations: First, the long-term efficacy of apigenin should be validated in chronic hyperoxaluric models, such as those induced by long-term high-oxalate diets or genetic hyperoxaluria, with particular attention to its preventive effects on interstitial fibrosis and stone recurrence. Second, given the relatively low oral bioavailability of apigenin, novel delivery systems should be developed to enhance its targeting and therapeutic efficacy. Additionally, exploring combination strategies with existing standard therapies (e.g., potassium citrate) and evaluating their synergistic effects and safety profiles are warranted. At the mechanistic level, further utilization of gene-edited animal models and structural biology approaches could clarify the interaction site between apigenin and AKT1 and elucidate downstream regulatory networks. Finally, advancing preclinical safety evaluations and conducting early-phase clinical trials, combined with biomarker validation in patient samples, will lay a solid foundation for the clinical application of apigenin as an adjunctive agent for kidney stone prevention.

## Conclusion

Overall, this study, for the first time reveals that apigenin alleviates calcium oxalate crystal-induced acute kidney injury by directly targeting AKT1 protein, thereby modulating two parallel signaling pathways – AKT1/mTOR and AKT1/FOXO3 – to synergistically exert anti-apoptotic and antioxidant effects. This finding not only deepens the understanding of the pathogenesis of nephrolithiasis but also provides important theoretical and experimental foundations for developing novel natural product-based strategies for kidney stone prevention. By implementing the systematic future research plan outlined above, we anticipate translating this discovery into innovative therapeutic approaches with clinical application potential.

## Data Availability

The data underlying this article are available on reasonable request to the corresponding author.
